# Evaluating digital maturity in public health systems: a statewide cross-sectional analysis of digital public health capacity in Missouri

**DOI:** 10.3389/fpubh.2025.1680904

**Published:** 2026-01-06

**Authors:** Anne Snowdon, Alexandra Wright, Abdulkadir Hussein, Josh Wymer, James Howgate, Tim Storey

**Affiliations:** 1 Odette School of Business, University of Windsor, Windsor, ON, Canada; 2 Missouri Department of Health and Senior Services, Jefferson City, MO, United States; 3 Guidehouse Inc, Chicago, IL, United States

**Keywords:** digital health, digital maturity, public health, health information systems, interoperability, healthcare quality, healthcare access

## Abstract

Digital transformation is critical to strengthening public health infrastructure, yet implementation across jurisdictions remains uneven. In the United States, state-level public health systems vary widely in their digital capabilities. Missouri’s Department of Health and Senior Services (DHSS) launched a digital modernization strategy to address these gaps. The objective of this work was to assess the digital maturity of Missouri’s public health system and examine how a validated digital maturity framework can inform strategic planning and investment. This was a cross-sectional, quantitative study using primary data collected from 59 public health teams across six DHSS divisions in 2024. The validated *Digital Health Indicator (DHI)* framework was applied via structured interviews to assess digital maturity across four dimensions: governance and workforce, interoperability, person-enabled health, and predictive analytics. Descriptive statistics and ANOVA were used to compare results across divisions, with forest plots used to visualize pairwise differences. The mean DHI score was 35.6/100, with substantial variation across divisions (range: 7–68). The Division of Community and Public Health achieved the highest scores (mean 50.8), while the State Public Health Laboratory scored lowest (mean 19.0). Interoperability and predictive analytics showed the greatest variability. Organizational readiness was consistently high, but constrained by limited infrastructure and uneven workforce digital training. A standardized digital maturity framework can reveal systemic strengths and gaps within public health systems, guiding targeted, specific, measurable, achievable, relevant, and time-bound (SMART) digital investments. Missouri’s experience demonstrates how structured assessment tools can support scalable and replicable digital transformation strategies applicable across U.S. states and other jurisdictions seeking to modernize public health services.

## Background

Digital transformation of public health systems has become increasingly critical for effective population health management and disease surveillance. Despite recognition of its importance, many public health systems are challenged by manual and paper-based processes (1, 2), with lack of information infrastructure with modern security features, data exchange capabilities, and few contemporary reporting tools (3). Digital health tools and technologies that support digitally-enabled health services have been identified as emerging digital drivers of health, highlighting the importance of data and digital maturity of public health systems to advance equitable and cost-effective approaches to public health service delivery (4, 5) In 2019, the Centers for Disease Control and Prevention (CDC) launched the Data Modernization Initiative (DMI), investing $50 million to transform public health infrastructure across the United States through enhanced technological innovation, data analytics, real-time data sharing, and automated reporting systems (1, 6, 7). This initiative aimed to improve timely and accurate data sharing to enhance evidence-based decision making in public health systems across the USA (8). While substantive fiscal investments are required to implement digital transformation, healthcare organizations need to be able to justify such investments and identify areas of digital strengths and opportunity to know where they need to plan and implement investments (9).

Digital health maturity evaluations measure healthcare system’s digital capacity, defined as the extent to which digital tools enable high-quality service delivery and care. In 2022, the Missouri Department of Health and Senior Services (DHSS) launched a five-year strategic plan that prioritized modernization of public health infrastructure, workforce capacity, and digital systems to strengthen population health (5). Within this context, assessing baseline digital maturity represents a critical first step to inform implementation planning and strategic investment. The objective of this study is to assess the digital capacity of the Missouri Department of Health and Senior Services (DHSS) and to examine how a standardized maturity framework can generate system-level insights to inform strategic planning. By applying a validated digital maturity framework across 59 DHSS program teams, this study offers a comprehensive analysis of digital strengths and gaps, aligned with Missouri’s modernization objectives. While digital health maturity has been studied extensively in clinical settings, U.S. public health systems rarely undergo standardized, program-level assessments. This study represents the first application of the HIMSS Digital Health Indicator (DHI) in a state public health system, offering actionable insights to guide statewide digital transformation.

Digital transformation in the United States has been advancing for many years. The Health Information Technology for Economic and Clinical Health Act (HITECH Act, 2009) fueled digital transformation with significant federal investments in health information technology through robust incentives spurring widespread adoption of electronic health records (EHRs) in clinical settings (10). However, while the Act successfully advanced EHR implementation in the clinical setting, advanced digital functionality and interoperability between clinical and public health systems has been slower to advance (8). While the CDC’s Data Modernization Initiative provides support for state-level digital transformation (8), without the use of validated, context-specific measurement tools, it is difficult for organizations to advance proactive, data-driven digital strategies that improve population health across diverse settings (9). While larger states like New York and California have successfully established statewide digital frameworks to enhance public health digital interoperability, their solutions are specifically designed for their unique contexts (11). Many states face significant constraints to support large-scale digital transformation including funding limitations (12), limited data governance frameworks, and clearly defined digital transformation pathways to inform implementation at scale for public health systems (2). The COVID-19 pandemic further exposed critical gaps in data capture and limited digital insight, restricting visibility into public health risks and documentation of outcomes (13).

These systemic weaknesses have highlighted the critical need for modernizing digital infrastructure to support disease monitoring, proactive interventions, and equitable care delivery (8). A number of public health organizations continue to face technical barriers such as inconsistent data standards, resource constraints, fragmented interoperability, and workforce capacity gaps that contribute to limited ability to securely share information across systems (2, 14). The impact of digital maturity extends beyond general operational improvements to specific public health interventions. Research consistently reveals that organizations achieving higher levels of digital maturity experience measurable improvements across multiple dimensions of healthcare delivery (30–32). Advanced digital capabilities, particularly in clinical decision support systems, have demonstrated direct positive effects on practitioner performance and population health outcomes (16). These systems illustrate how digital maturity can transform public health practice by providing real-time guidance and enhancing disease surveillance, ultimately improving both individual and community health outcomes. The gap between current capabilities and digital transformation highlights the importance of measuring the current state of digital maturity for public health to objectively inform strategic planning to advance digital transformation in public health systems.

Recent public health challenges have highlighted the urgent need for digital transformation to strengthen and automate disease surveillance, proactively identify risks to the health of the public, and to strengthen public health services and strategies that sustain and advance population health. There is significant evidence emerging that supports the use of digital tools to uphold the core principles of public health, while delivering proactive public health services uniquely tailored to community needs (4, 17). Digital technologies have been identified as the next determinant of health (18), highlighting the importance of data and digital maturity of public health systems for more equitable and cost-effective approaches to public health services (4).

Digital maturity can be highly varied across healthcare organizations or even within the same system. The uneven development of digital capabilities within the same organization, as frequently observed in public health agencies, follows distinct maturity patterns identified in organizational change literature, where leadership commitment, resource allocation, technical infrastructure, and workforce capabilities support or hinder environments for digital advancement (19, 20). Current digital health assessment frameworks demonstrate significant variations in scope and application, with most tools focused on national or global assessment rather than providing actionable measurement capabilities for individual public health agencies. Existing frameworks tend to either focus on specific technological components (such as analytics or interoperability) or attempt system-level assessment creating a gap in validated, multidimensional tools that assess organizational readiness for digital public health transformation (5, 21–23).

Analysis of digital maturity variations across organizations can be understood through both technological and organizational lenses. The uneven development of digital capabilities within the same organization, as observed in this study, aligns with organizational change theories that emphasize how leadership commitment, resource allocation, historical funding patterns, and implementation affect digital adoption rates across different functional units (19). This theoretical perspective helps explain why some divisions within the same public health agency might achieve substantially different levels of digital maturity despite operating under the same organizational leadership.

Currently, there remains a critical gap in standardized assessment methodologies to document digital maturity (24) and digitally-enabled public health program evaluations (25). Recent frameworks (26), propose public health digital assessment dimensions but lack practical validation and detailed measurement criteria. International consensus work, cited from (15), has also outlined core technological capabilities required to assess digital excellence across high-performing health systems (15), offering further support for structured digital maturity evaluation approaches. The Digital Health Indicator (DHI) was selected for this study based on its multidimensional framework and documented use in health system settings. Existing peer-reviewed studies have examined the tool’s construct validity, internal consistency, and practical utility in differentiating digital maturity levels across healthcare organizations (9, 27, 28). While primarily used in clinical contexts, its application here explores its relevance for public health systems, recognizing the potential limitations in cross-sector transferability. The DHI tool has been widely adopted across the globe to measure digital maturity of health organizations, providing important insights into the progress of digital transformation (9, 27, 28). The DHI provides a comprehensive online survey tool that has been globally validated to measure the progress made on the four dimensions of digital transformation; interoperability, person-enabled health, predictive analytics, governance and workforce. The DHI framework was designed to evaluate digital health capabilities across all types of health systems, including public health agencies, making it available for adaptation for assessing state-level public health digital maturity with minimal adaptation required. In addition to documenting digital capacity, this study advances methodological rigor by applying a validated tool to the measurement of digital maturity in the public health context. The DHI tool offers a framework for measuring public health digital capacity and maturity to guide evidence-based digital transformation for public health systems.

Numerous frameworks exist for assessing digital health maturity at national, organizational, and workforce levels, including the WHO’s Global Digital Health Index (17), the World Bank’s Digital Health Assessment Scoring Tool (21), and various competency models targeting health worker digital literacy (22). However, many of these models focus on macro-level benchmarking or assess only specific components, such as infrastructure or interoperability, limiting their direct applicability to program-level assessments in state public health systems. The Digital Health Indicator (DHI) was selected for this study due to its multidimensional structure and capacity to assess digital readiness at the organizational and team level. While developed for broader healthcare use, the DHI’s four domains: governance and workforce, interoperability, person-enabled health, and predictive analytics, closely align with the Missouri DHSS’s stated objectives to build a digitally enabled workforce, strengthen system interoperability, expand digital services for citizens, and enhance use of real-time data to inform decision-making (6). Moreover, the DHI was available through an existing partnership with HIMSS, allowing for full implementation across all 59 teams. While this study does not present a formal logic model, the alignment between the DHI domains and DHSS’s digital priorities provided a practical and conceptually appropriate rationale for its use in this context.

## Methods

### Study design

**Figure 1:**
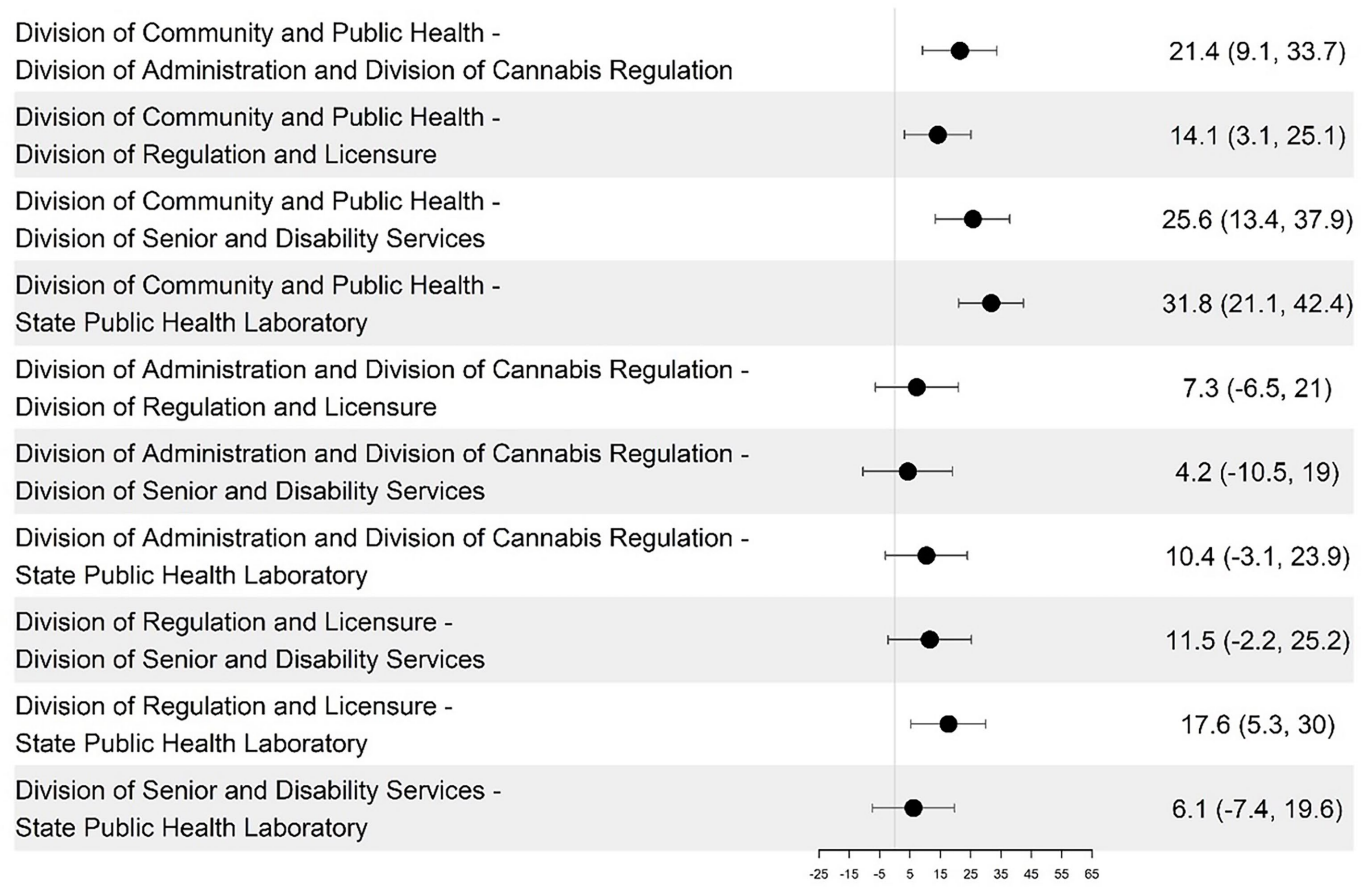
Differences in mean DHI scores among the divisions and their 95% confidence intervals.

A standardized digital maturity assessment tool was used to measure public health digital capacity. The Digital Health Indicator (DHI) tool was chosen for digital maturity assessment due to its use across global health systems and validation in the literature (9, 26). While originally developed for clinical settings, the tool was applied here with minor adaptations to align with public health terminology and context. The DHI measures digital maturity across the ecosystem of health services that connects clinicians and provider teams with individuals (in this study, citizens), enabling self-management of health and wellness using digital tools in a secure environment. The framework has been examined empirically in multiple studies (9, 28), and was designed to be outcomes focused, agnostic to technology, and aligned with the quintuple aim health system performance outcomes (29). There are four dimensions measured in the DHI, including governance and workforce, person-enabled care, predictive analytics, and interoperability. The concepts and features of the Digital Health Indicator model are illustrated in Figure 1 and described in detail in a HIMSS publication (29). The online DHI assessment includes 120 indicators scored on a five-point Likert scale across four dimensions, yielding a total possible score of 400.

### Setting and participants

In this study, the DHI was employed to measure digital health capacity among 59 public health teams within a state public health system. While the DHI was designed to be used for all global health systems, including public health, it was applied in this study with minimal changes to terminology, while maintaining the validated structure and dimensional framework of the original tool. This approach preserved measurement validity while enhancing relevance for public health professionals responding to the assessment. The research team ensured that all modifications enhanced content and face validity for the public health context without altering the underlying logic, item structure, or psychometric integrity of the tool. This approach ensured that the DHI maintained fidelity to its validated structure while supporting contextual relevance to the State Public Health context. The DHI’s application in this study was informed by previous peer-reviewed evaluations confirming its reliability, construct validity, and applicability across system types (9, 28). To ensure its framing was recognized within the Missouri public health system, an in-depth review of the DHI indicator statements, each one accompanied by a public health example, was conducted to ensure alignment and relevance within the unique context of Public Health. The review involved two Public Health experts who examined terminology and contextual appropriateness of each of the 120 DHI indicators, resulting in minor terminology changes to approximately 30% of the indicators (ex. “patient” was changed to “citizen”) to further support clarity of each question item within the public health context. The two reviewers independently reviewed each indicator and example, and 100% agreement was reached for all indicators. The terminology changes focused on contextual relevance while preserving the validated measurement structure. Examples of indicator changes included changing patient monitoring to “disease outbreak monitoring” and changing “health organization” to “public health organization” to ensure relevance for public health professionals. The review process maintained the construct validity of the tool while enhancing the face validity of the DHI tool for public health contexts. The public health version of the DHI was pilot tested in two phases. The first phase consisted of a comprehensive review with the public health expert guiding the implementation of the Missouri DHSS project. This validation phase critically reviewed each indicator and accompanying examples to ensure consistency and accuracy of the DHI tool’s validity for DHSS project objectives. Following incorporation of this feedback, a second test was conducted with DHSS senior leadership to validate the clarity, alignment, and contextual relevance of all 120 indicators for Missouri’s public health system.

Missouri leaders introduced the 59 teams to the research team and invited each team to participate in the project, and directed the process for researchers to contact each team. The project plan was reviewed by the Chair of the IRB who determined that IRB review was not required. The 59 teams were divided into five groups representing six DHSS Divisions including: State Public Health Laboratory (SPHL) (n = 12), Division of Community and Public Health (DCPH) (n = 20), Division of Regulation and Licensure (DRL) (n = 11), Division of Senior and Disability Services (DSDS) (n = 8) and Division of Administration (DA) (n = 6) and Division of Cannabis Regulation (DCR) (n = 2). The Division of Administration and Division of Cannabis Regulation were combined (n = 8) due to their small individual sample sizes and shared operational profiles as non-client-facing, administrative groups. This consolidation allowed for more robust statistical comparisons while acknowledging their limited engagement in direct public health service delivery.

### Procedure

All participating public health teams across Missouri DHSS programs were invited by email to participate in the project by their executive leadership across their Divisions, Bureaus, Offices, Programs, and Units. The research team managed logistics and scheduling of guided interviews with each team to complete data collection. All teams that offered public health services to Missouri residents completed a full DHI assessment, including all four dimensions—Governance and Workforce, Interoperability, Predictive Analytics, and Person-Enabled Care. Those participating programs whose mandate and roles did not engage Missouri residents directly (ex. administration, laboratory) completed a partial DHI assessment that did not include the Person-Enabled dimension as these teams had no direct engagement with Missouri citizens. Two of the teams worked very closely together and requested a joint guided interview to complete the DHI assessment. The total number of DHI assessments completed was 59.

### Data collection

Guided interviews were conducted to administer the DHI survey tool via Microsoft Teams over a five-week period from September to October 2024, with interviews ranging from 60 to 120 min. Interviews were recorded with participant consent to enable researchers to review responses for clarity and accuracy. During guided interviews, researchers administered the 120 DHI indicator statements, and recorded participant responses on the five-point Likert scale from ‘not enabled’ to ‘fully enabled’ for each indicator. The guided interview strategy enabled a structured and consistent approach to data collection across all 59 teams while accommodating the unique context of each program’s operations.

Responses to survey items were transcribed onto an online survey platform which automatically generated a DHI score for each team. Data collection was completed during a 2-h online meeting with each team. To minimize workplace disruption, some interviews were completed across two sessions of approximately 1 h in duration.

This project plan was reviewed by the Chair of the Institutional Review Board (IRB) for the State of Missouri Department of Health and Senior Services (DHSS) who determined the project did not require IRB review given the focus on program evaluation and no PII data would be collected.

### Analysis

To conduct the quantitative analysis, DHI scores were first computed as a percentage of the points achieved out of the total points possible for each dimension. The DHI measures were all converted into percent scale to allow for comparisons across a standardized scale (0–100). This method made it possible to account for indicators that were not relevant to a particular participating unit (ex. person-enabled indicators), because the points pertaining to that indicator were removed from the total points achievable. Descriptive statistics and Analysis of Variance (ANOVA) were used to identify statistically significant differences among units and divisions. The pairwise comparisons between each pair of programs were illustrated using forest plots, which show estimates of the differences as horizontal bars that represent 95% confidence intervals with estimated difference as their midpoints. If the horizontal bars demonstrating the comparison do not intersect with the vertical line of zero then the differences in score are statistically significant.

A comparative analysis of DHI results across the DHSS teams was completed to identify digital strengths, gaps and variability in results across the 59 teams, for each of the four dimensions of digital transformation. This analysis examined the DHI scores for each group of the teams to identify and compare areas of strengths in digital capacity, and gaps in digital maturity across the State public health system. The comparative analysis examined dimension level scores for each of the teams, to identify patterns in the strengths and gaps in digital capacity for each of the four dimensions measured by the DHI. Key areas of strengths and gaps in digital capacity were analyzed to inform leaders of opportunities to leverage key strengths to inform and advance their digital transformation strategy for the State public health systems. This comparative analysis was designed to reveal aspects of digital health maturity that was consistently observed across multiple programs and divisions based on their DHI scores. Finally, a report was produced integrating the findings of the comparative analysis with the quantitative results to provide context for the statistical variations observed across the 59 teams participating in the study.

## Results

### Department DHI averages by division and dimension

**Figure 2:**
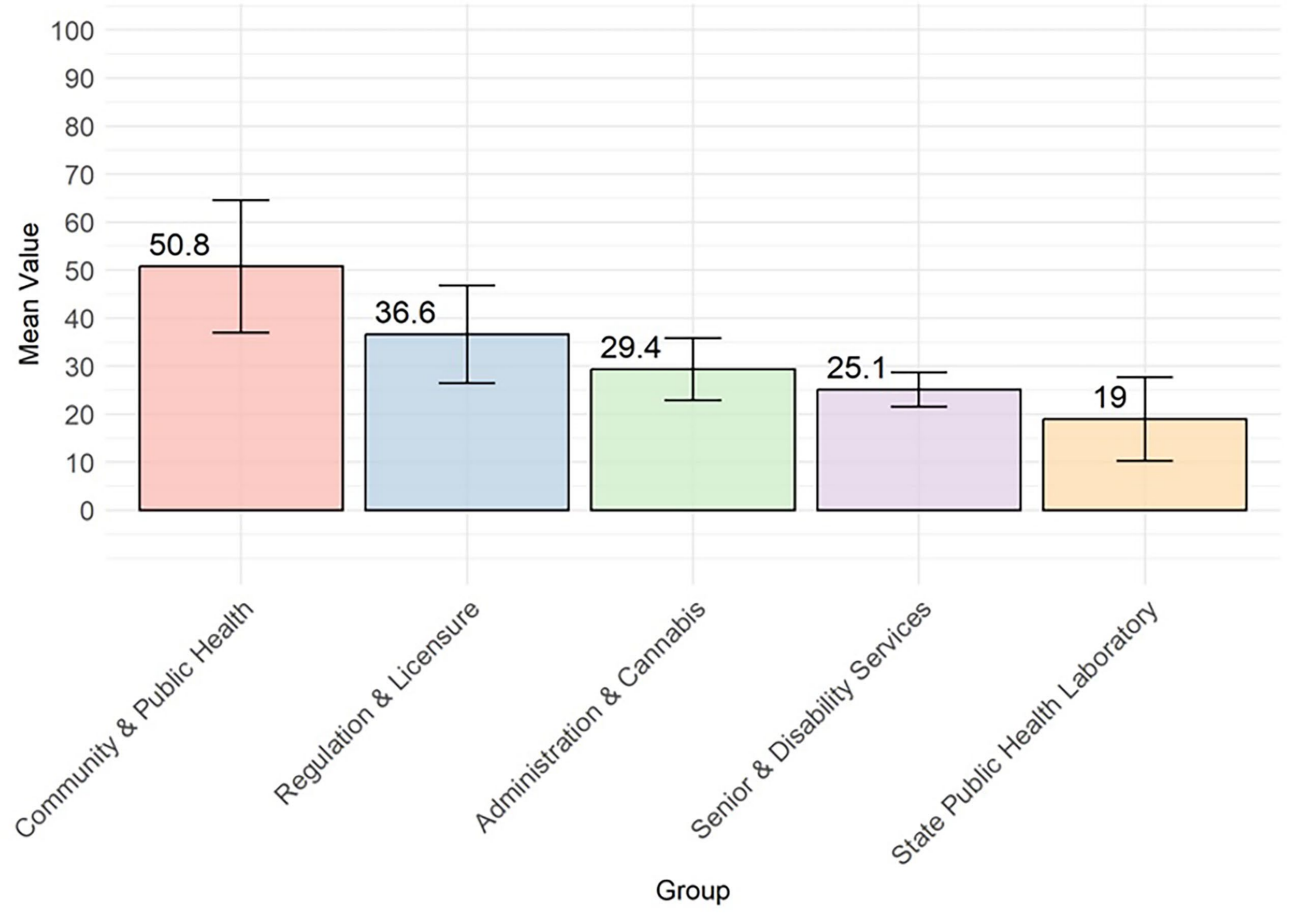
Mean and standard deviation error bars for the overall DHI by division.

The following highlights the quantitative results from the DHI assessments. The overall mean DHI score for the DHSS was 35.6 (Standard Deviation = 16.1). This DHI index score is a summative average across all 59 program scores, calculated as the percentage (out of 100) achievement for each of the four dimensions of the DHI (Table 1; Figure 1). DCPH achieved the highest average score of 50.8, followed by DRL (36.6). The lowest average was 19.0 for SPHL (Figure 2).

**Table 1:** Department DHI averages and ranges by division and dimension compared to the North American (NA) average.

**Division**	**DHI score** **(mean)**	**Governance and workforce** **(NA mean=60/100)**	**Interoperability** **(NA mean=75/100)**	**Person-enabled** **(NA mean=50/100)**	**Predictive analytics** **(NA mean=40/100)**
Mean scores	35.6	52.5	62.2	37.4	24
Range of scores	7-68	9-95	11-95	6-82	0-85
Division of Community and Public Health (DCPH)	50.8	65.6	84.9	54.9	46.2
Division of Regulation and Licensure (DRL)	36.6	55.8	79.1	N/A	12.3
Division of Administration and Division of Cannabis (DCR)	29.4	43.5	51.0	N/A	19.1
Division of Senior and Disability Services (DSDS)	25.1	45.5	39.6	30.8	4.8
Division of State Public Health Laboratory (SPHL)	19.0	37.2	32.0	14.4	17.8

**Figure 3:**
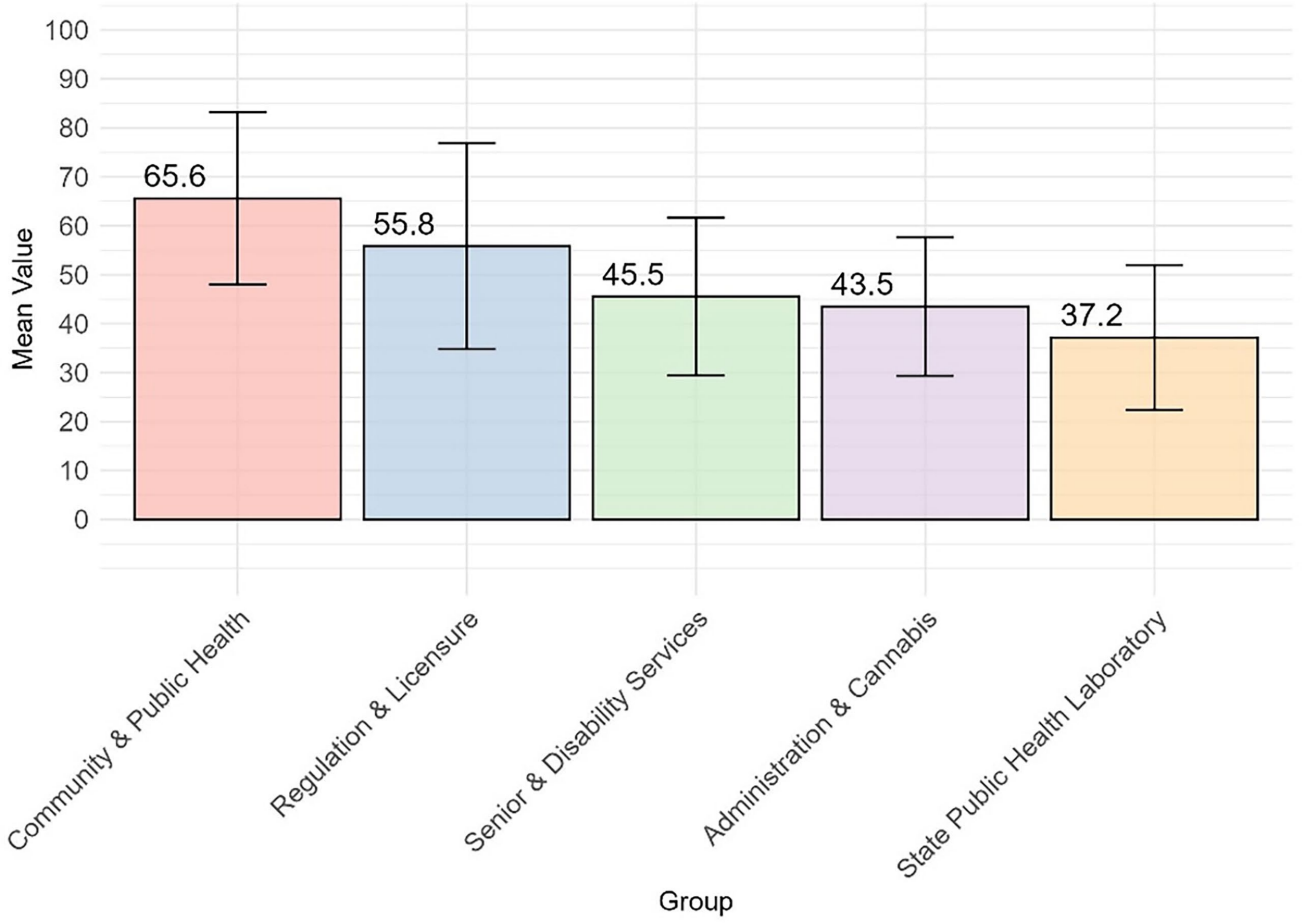
Mean and standard deviation error bars for the overall governance and workforce dimension by division.

**Figure 4:**
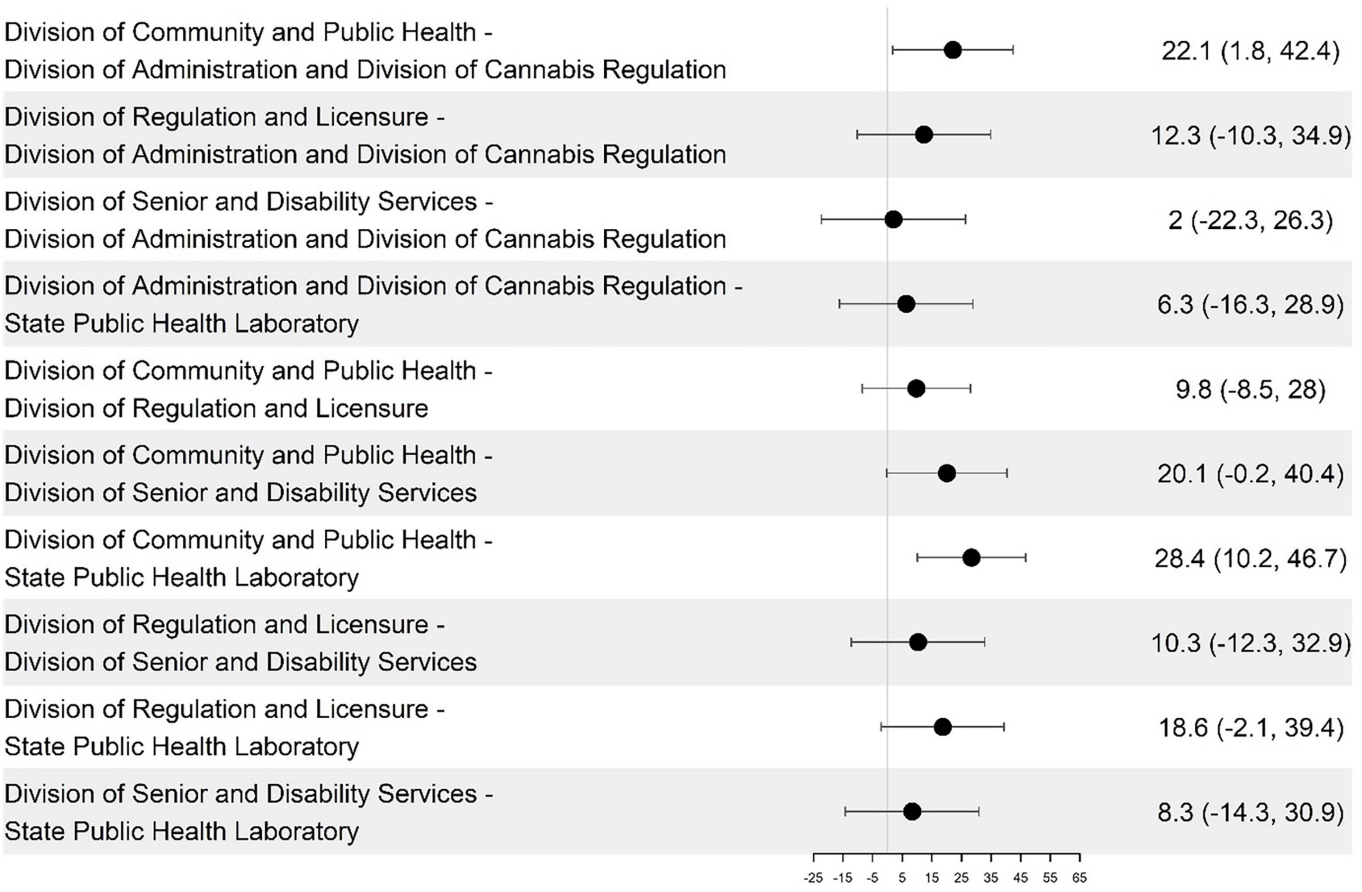
Pairwise differences in mean governance and workforce dimension scores among the divisions and their 95% confidence intervals.

The DCPH had a consistently higher average DHI score than the other groups (14–32 points higher), and SPHL had a significantly lower average score. However, the differences between the remaining group pairs were not statistically significant (Figure 1). The DHSS divisions were analyzed and compared across the four DHI dimensions of governance and workforce, interoperability, predictive analytics, and person-enabled health. For the governance and workforce dimension, DCPH scored higher than DA and DCR by 22.1 points (*p* = 0.021), and DCPH is higher than SPHL by 28.4 points (*p* = 0.0004) (see Figures 3, 4; Table 2). The remaining differences among public health teams were not statistically significant.

**Table 2:** Governance and workforce sub- dimension scores by division (scored from 1 to 100).

Division	Policy and decision-making	Stewardship	Workforce Capacity	Transparency
Community & Public Health	58	63	68	64
Regulation and Licensure	55	46	62	38
Administration and Cannabis	42	39	51	37
Senior & Disability	37	38	41	29
Laboratory	33	34	39	23

**Figure 5:**
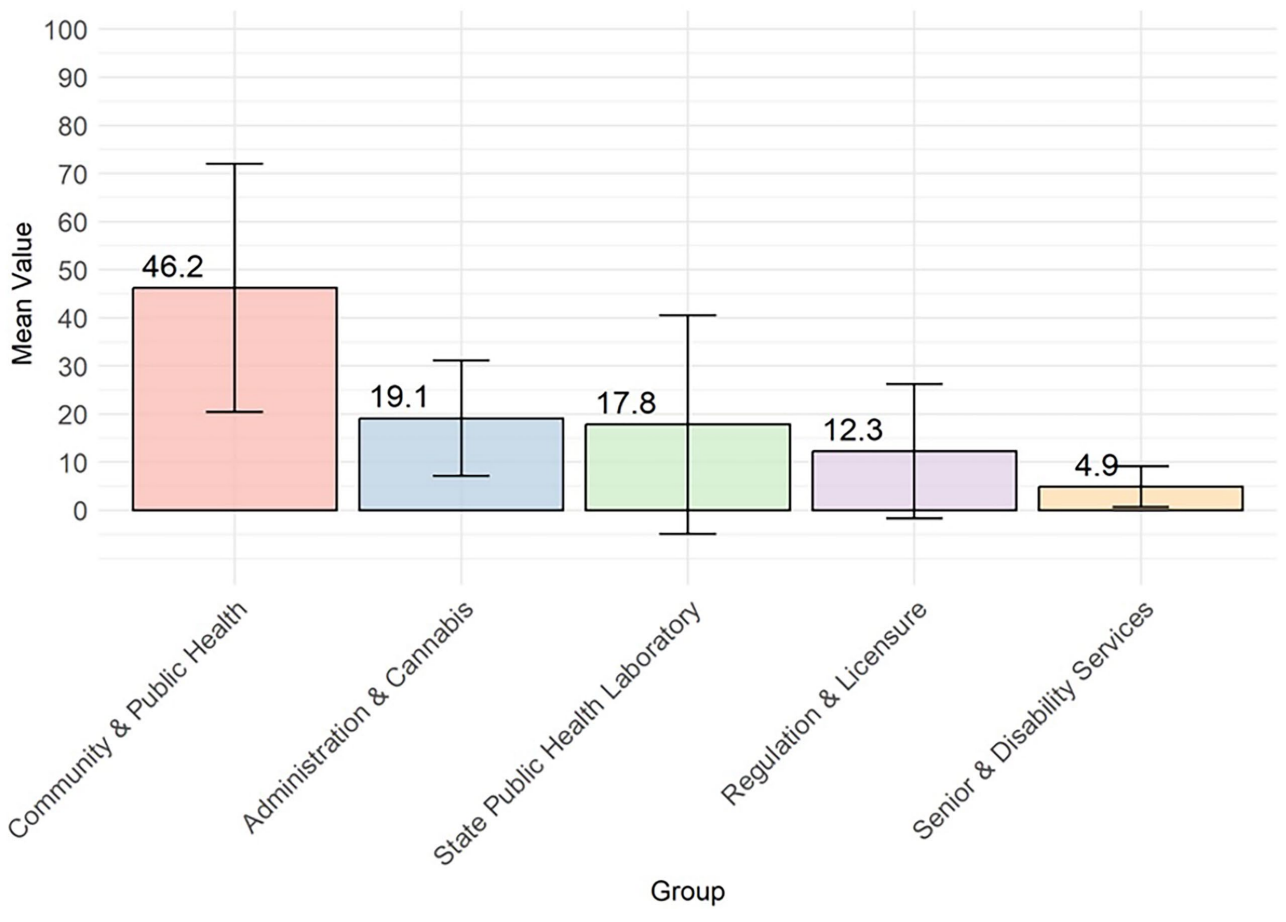
Mean and standard deviation error bars for predictive analytics by division.

**Figure 6:**
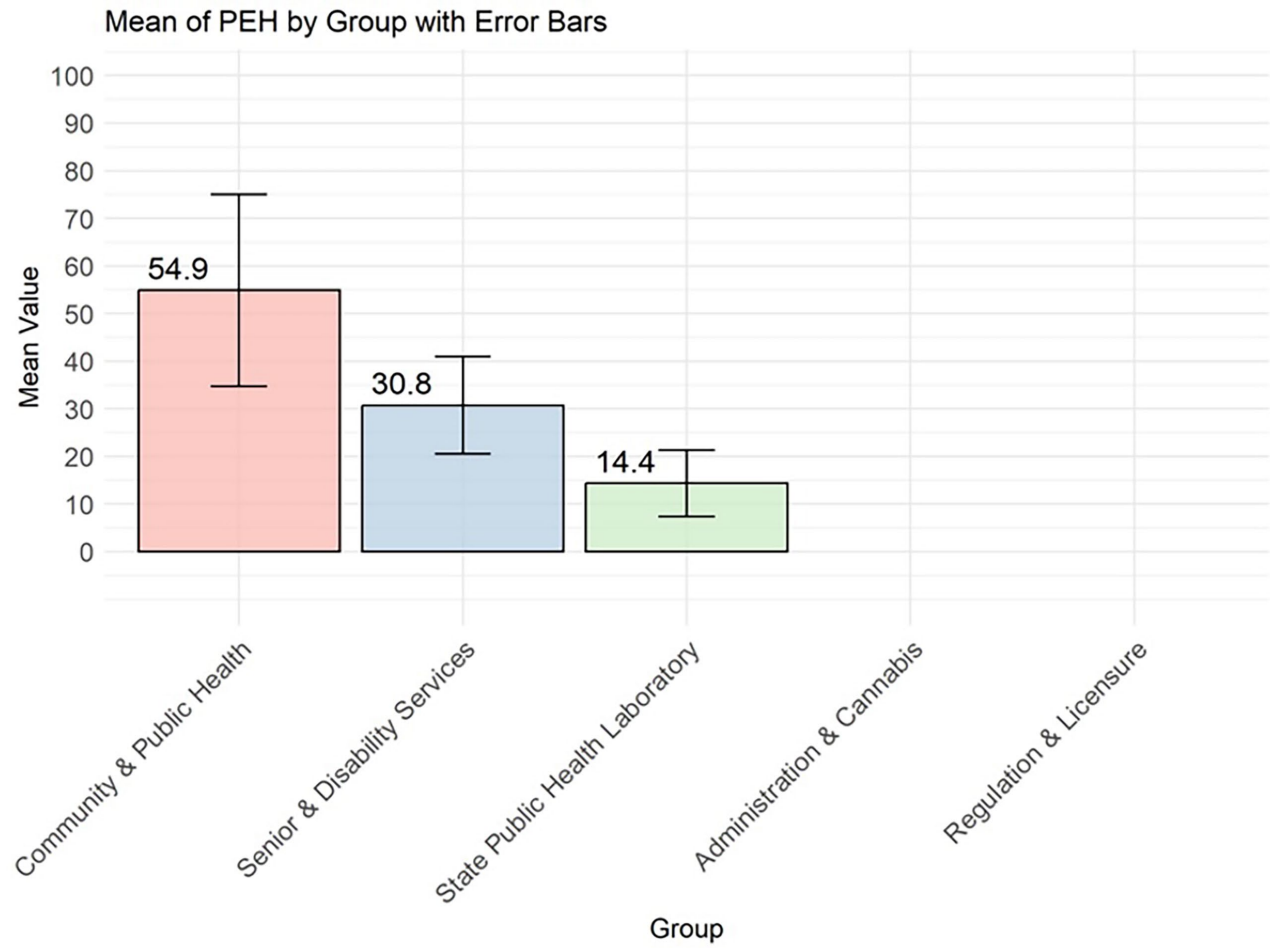
Mean and standard deviation error bars for person-enabled health by division.

The interoperability dimension results revealed significant interdepartmental variations. DCPH demonstrated exceptional scores on interoperability, exceeding DA and DCR by 17.76 points (95% CI, *p* < 0.0001), SPHL by 32.22 points (95% CI, *p* < 0.0001), and DSDS by 26.11 points (95% CI, *p* < 0.0001). DRL surpassed SPHL by 25.86 points (95% CI, *p* < 0.000) (Figures 5, 6).

**Figure 7:**
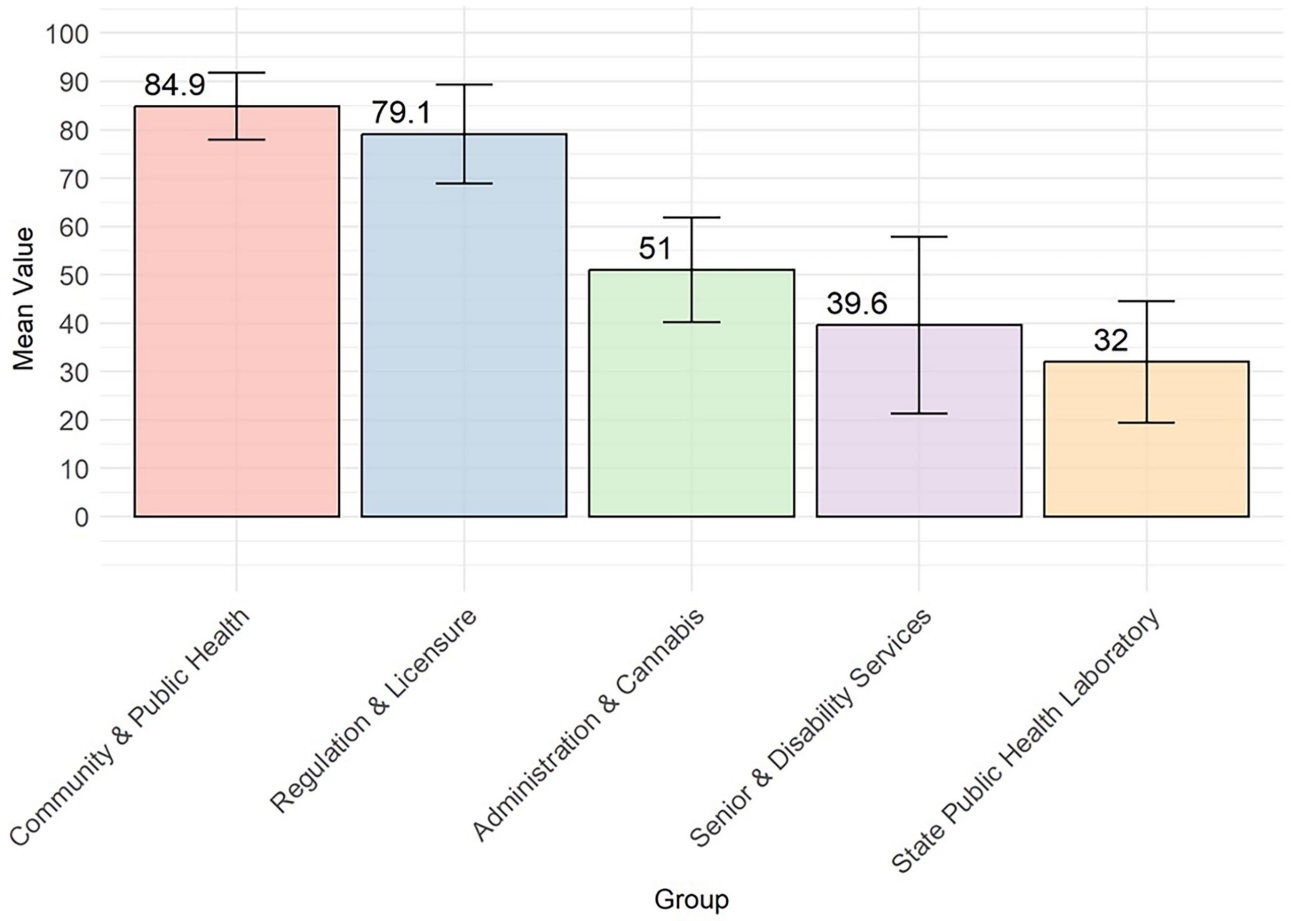
Mean and standard deviation error bars for the overall interoperability dimension by division.

**Figure 8:**
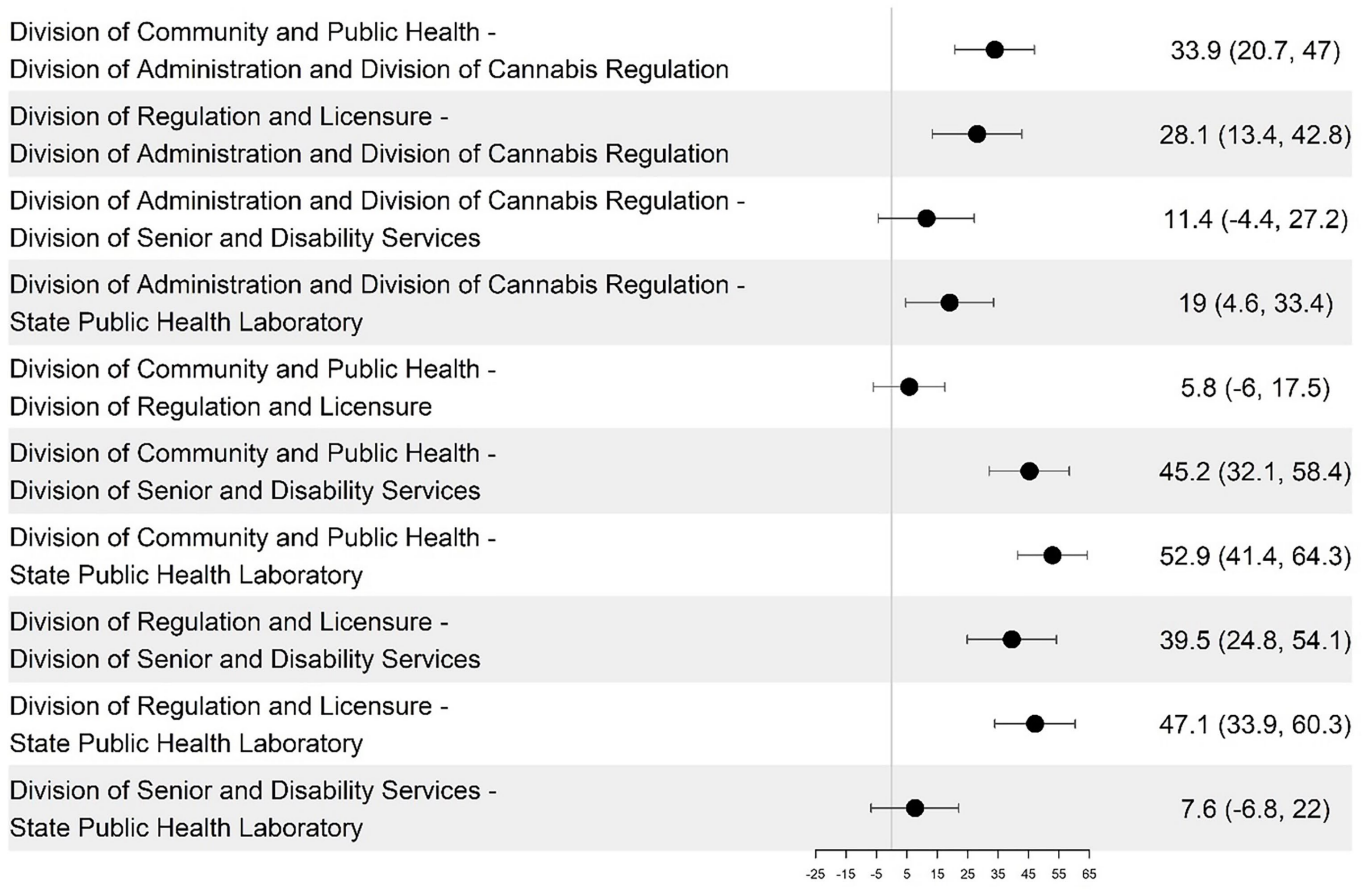
Pairwise differences in mean interoperability dimension scores among the divisions and their 95% confidence intervals.

Predictive Analytics capabilities varied substantially across divisions (Figures 7, 8). DCPH achieved the highest scores across operational analytics (40/100), personalized analytics (39/100), and predictive analytics (36/100) categories (Figure 5; Table 3) DCPH significantly outperformed all other divisions on predictive analytics, exceeding the Division of Senior and Disability Services (DSDS) by 41.3 points (*p* = 0.00002) and the State Public Health Laboratory (SPHL) by 28.4 points (*p* = 0.00002). The Division of Regulation and Licensure (DRL) results revealed some progress for operational analytics capability (24/100) but limited personalized (4/100) and predictive (8/100) analytics capacity (Table 3). The Divisions of Administration and Cannabis Regulation reported similar scores for operational, personalized, and predictive analytics, respectively, (30/100, 8/100, and 17/100) (Table 3), indicating limited analytics capability. DSDS and SPHL demonstrated the lowest maturity across all analytics subdomains. Consistent with overall DHI results, DCPH was the only division to demonstrate analytics capability across all three subdomains.

**Table 3:** Predictive analytics scores by division (scored from 1 to 100).

Division	Operational	Personalized	Predictive
Community & Public Health	40	39	36
Regulation and Licensure	24	4	8
Administration and Cannabis	30	8	17
Senior & Disability	14	3	4
Laboratory	17	1	7

**Figure 9:**
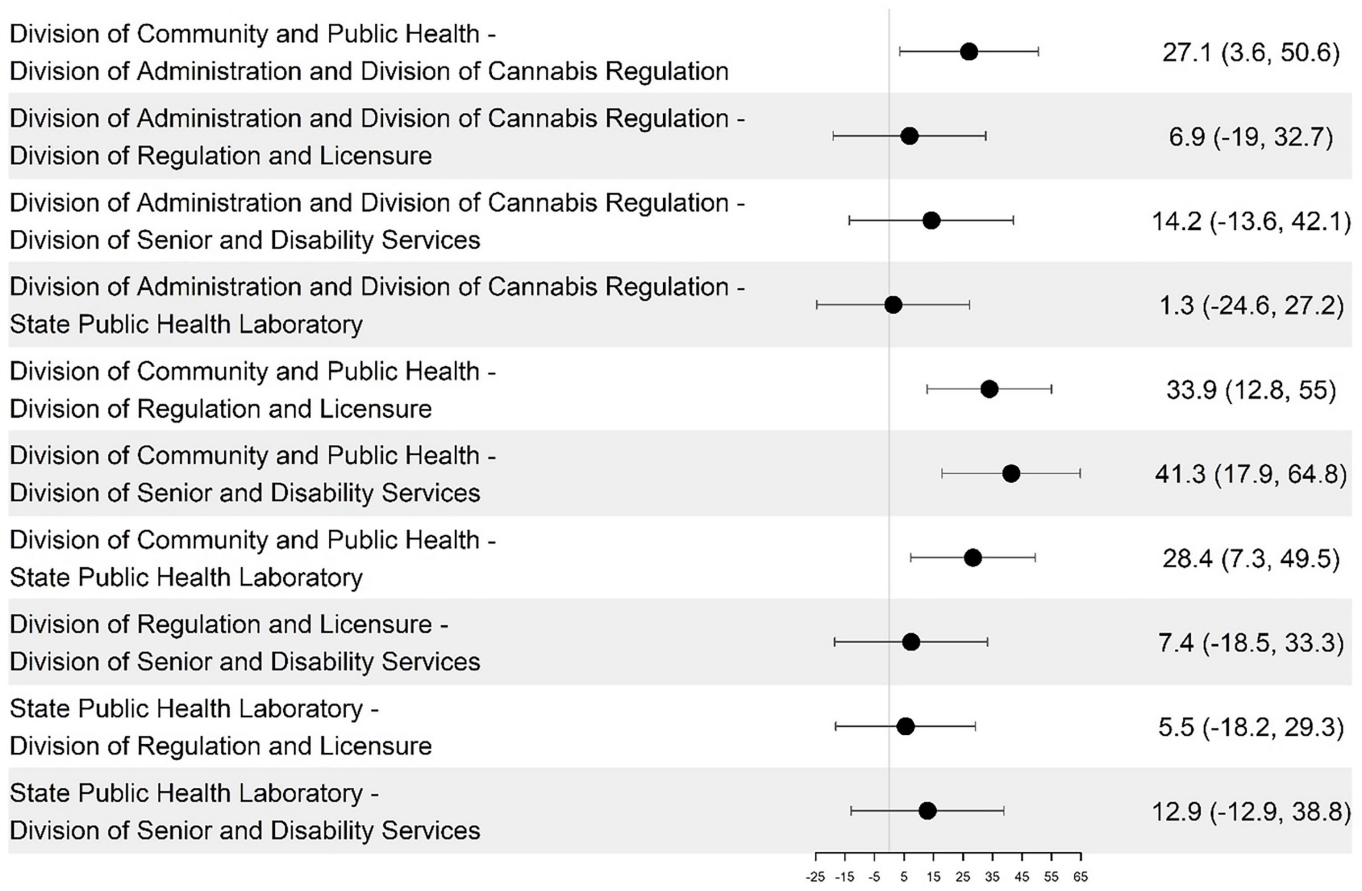
Pairwise differences in mean predictive analytics dimension scores among divisions and their 95% confidence intervals.

Person-Enabled Health scores revealed significant differences across teams, with DCPH exceeding DSDS by 27.19 points (95% CI, *p* = 0.015) and SPHL by 45.96 points (95% CI) (Figure 9; Table 4). DA, DCR, and DRL were excluded from this analysis as non-client-facing organizations. When comparing the 59 programs, the DCPH outperformed all of the others on all four dimensions of the DHI. This is particularly the case when DCPH is compared to DA and DCR, DSDS, and SPHL (Table 1).

**Table 4:** Person-enabled scores by division (scored from 1 to 100).

Department	Personalized	Predictive	Proactive
Community & Public Health	39	43	35
Regulation and Licensure	N/A	N/A	N/A
Administration and Cannabis	N/A	N/A	N/A
Senior & Disability	19	13	9
Laboratory	11	10	6

### Comparative analysis results

The comparative analysis summarizes observed differences in DHI scores across divisions and programs. There were significant variations in digital infrastructure and interoperability capabilities across the 59 teams. Teams within the DSDS, SPHL, and the Division of Administration (DA) had lower interoperability scores for each sub-dimension related to teams reporting “minimally enabled or not enabled” access to digital tools, integration with public health records, or ability to exchange data with external health agencies (e.g., federal health agencies) (Figure 7). For example, many programs reported no automated or secure data exchange with other public health agencies or required federal reporting systems. In contrast, programs within the DRL and several DCPH areas, such as Maternal Child Health, HIV, STD and Hepatitis, and Vital Records, reported advanced levels of interoperability and digital connectivity, including the ability to integrate social determinants of health (SDOH) data into their program level information systems. Across all divisions, all 59 teams consistently demonstrated advanced privacy and data security capacity, which included encrypted data-sharing protocols and department-wide compliance with state-level privacy standards, consistent with higher scores on security-related indicators (see Figure 8).

Program teams across all DHSS divisions consistently demonstrated high scores on organizational support for data-informed decision making, digital literacy, and workforce learning (Figure 3). Teams scored lower on indicators related to formal upskilling programs and partnerships with academic institutions to support digital competency development, with DCPH scoring higher than other divisions.

**Figure 10:**
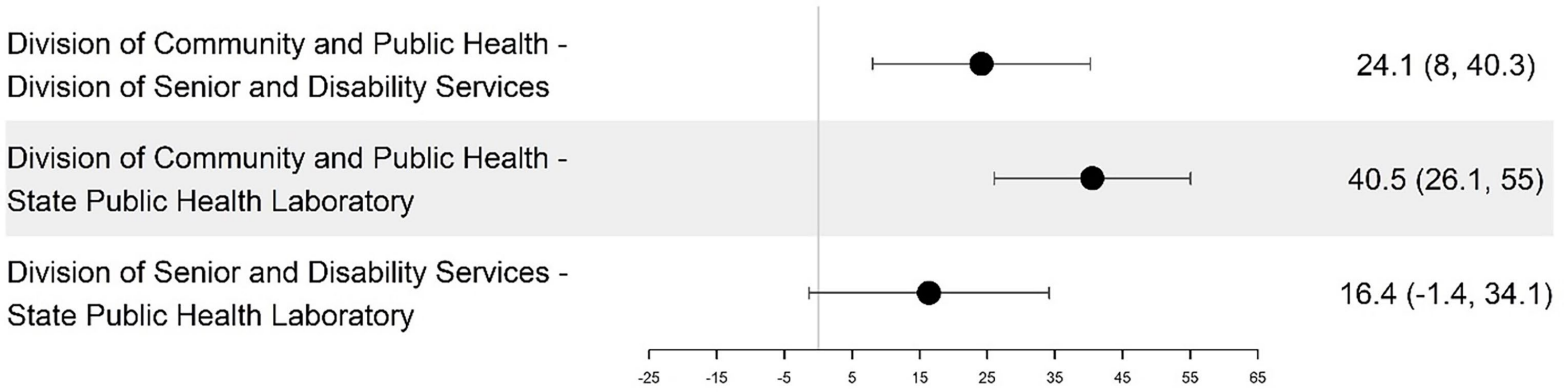
Pairwise differences in mean person-enabled dimension scores among the divisions and their 95% confidence intervals.

Among DHSS programs that deliver direct public health services to citizens (DCPH, DSDS, and SPHL), person-enabled digital maturity scores demonstrated significant variation, with DCPH outperforming DSDS by 27.19 points (95% CI, *p* = 0.015) and SPHL by 45.96 points (95% CI, *p* = 0.000009) (Figures 9, 10). Quantitative results showed person-enabled health was the lowest-scoring dimension across divisions and significantly lagged governance and interoperability scores (Table 1). Consistent with these findings, many programs reported no patient-facing portals, virtual services, or digital mechanisms for viewing test results or public health records, resulting in continued reliance on paper-based processes. The SPHL, Protective Services, and Environmental Epidemiology units reported no capacity for citizens to access individual records, indicating substantial service limitations. A small number of programs, including WIC, Maternal Health, and Immunizations, demonstrated more mature digital infrastructure and the ability to connect to clinical data systems; however, these were exceptions. Together, these results indicate that digital engagement of citizens remains at an early stage, with capabilities concentrated in isolated programs rather than system-wide (see Figures 9, 10).

## Discussion

This study presents the first known comprehensive assessment of digital health maturity across a state public health system using a structured measurement framework to examine governance and workforce, interoperability, predictive analytics, and person-enabled care. These findings offer timely insights as Missouri DHSS advances its public health infrastructure, providing an evidence base to inform decisions on digital investments and system design. The results demonstrate that Missouri’s DHSS is advancing the foundational strengths to advance digital transformation, with programs consistently demonstrating leadership commitment to advance data-driven decision making and support for workforce development. The findings suggest there is an organizational readiness to advance digital transformation which is a critical enabler of digital transformation and represents a notable strength in a public health context where resistance to change is often cited as a major barrier. Prior research (1, 2) has emphasized the importance of leadership engagement and cultural readiness in successful digital transformation efforts. The findings suggest that DHSS has already achieved the crucial first step by establishing a system-wide culture of readiness that values evidence, and the strategic use of digital tools to advance public health services delivery.

The comparative analysis revealed substantial variation in digital maturity across DHSS divisions, reflecting uneven progress in advancing interoperability, workforce enablement, and analytics capability. Divisions such as the DCPH achieved higher maturity due to established interoperability frameworks and integration of social determinants of health data, while divisions such as SPHL and DSDS demonstrated limited use of analytics and interoperability functionality. These differences illustrate high variation in digital infrastructure advances across the Missouri Public health teams, resulting in significant differences in digital capacity among teams within the same State public health system. Although organizational readiness and leadership commitment were consistently strong across all teams, the absence of structured workforce training programs and inconsistent access to analytic tools limited the progress of digital transformation. Person-enabled health scores were notably lower than all other dimensions of digital transformation for most divisions, which suggests there is limited capacity for citizens to access or manage their own health information, and digitally enabled services that engage citizens with public health teams remain underdeveloped. High variation in digital capabilities for public health teams may have implications for equitable access to public health programs, particularly in rural and high-need communities.

The capacity for interoperability across the 59 teams revealed substantial variations in digital infrastructure which may create a “digital divide” where some programs can effectively exchange data with clinical systems, digital programs, and teams while others lack access to basic digital systems. This fragmentation not only limits coordinated public health response, but also introduces inequities in service delivery, where access to timely, data-informed interventions may depend on which program or division a citizen has access to or engages to seek public health services. In effect, the digital divide risks becoming a driver of population health disparities, especially for rural or underserved populations where public health programs are less digitally mature. These patterns are consistent with prior research (9, 12) indicating that digital maturity is a key determinant of health system performance, quality, and workforce outcomes. Programs including Women, Infants and Children (WIC), Epidemiology, and Maternal Health were able to effectively analyze health trends in real-time and were able to connect social determinants data with clinical outcomes, to identify population health risks before they escalate to crisis levels. The capacity for predictive analytics enables public health teams to advance data driven decisions that shift services from reactive to proactive service delivery, directly supporting Missouri’s public health mandate. Conversely, programs with limited analytics capabilities remain constrained by their lack of analytics capacity to accurately forecast or identify risks to inform preventive measures that mitigate emerging health risks for communities or populations.

Variability in the capacity for interoperable flow of data among public health teams was evident in this study and has significant implications for advancing public health services. Limitations in interoperability are associated with data silos that limit the flow of data to inform decisions such as program decisions without the benefit of shared insights or coordinated action, as it prevents analysis of data across programs that is needed to address complex public health challenges that span multiple service areas. During disease outbreaks or other public health emergencies, programs with limited interoperability or analytics capabilities face substantial challenges in disease monitoring, timely detection of emerging threats, and coordinated response planning. The inability to integrate clinical and population health data in real-time can delay identification of outbreak patterns, impede targeted intervention strategies, and ultimately compromise the effectiveness of public health response efforts, particularly for vulnerable populations at highest risk.

The capacity for supporting citizens with digital tools and devices to manage their health and report or access personal health information, was very limited across DHSS teams signaling this as a key opportunity to advance public health services in Missouri. These findings support Kickbusch’s (8) framework describing the digital divide as a determinant of inequity, where programs with advanced digital maturity offered citizens multiple access channels, including virtual services and digital health management tools, particularly beneficial for rural communities and vulnerable populations facing barriers to in-person services. The highly varied digital capabilities across DHSS programs raises important equity considerations for public health services delivery. Research (4) identifies digital technologies as determinants of health that can either advance or impede equitable outcomes. The limited capacity for person-enabled digital public health services that some DHSS programs experience may disproportionately affect populations with barriers to access, including rural communities and economically disadvantaged groups. Studies (12) suggest that strategic digital investment must prioritize the development of both internal capability and external digital accessibility to ensure digital transformation efforts advance, rather than compromise, health equity objectives for communities and populations. Digital technologies have been established (4) as key determinants of health (18) outcomes that influence service equity and access. Across the DHSS, identified challenges in person-enabled digital services for some programs may further limit access to public health services for unique population groups or communities.

This study demonstrates that digital maturity assessment provides crucial visibility into organizational strengths and weaknesses to inform decisions on mobilizing resources to achieve targeted outcomes and advance digital public health services. Organizational readiness and leadership support, while necessary, are insufficient without corresponding information infrastructure and investments in workforce training, a key insight for systems in early phases of digital transformation. Validated tools to assess digital maturity, like the DHI, offer health leaders insights and an actionable roadmap to guide investment, align resources, and bridge the digital divide.

As public health systems face growing demands for equity and public health responsiveness, objectively assessing digital assets, strengths and gaps are critical for informing leadership decisions and strategies to advance digital transformation at scale. This comprehensive approach to measuring the four dimensions of digital transformation offered insights into both technological infrastructure and organizational readiness factors that may enable and empower progress towards digital transformation of public health services. This study demonstrates that validated assessment tools to measure digital capacity and progress towards transformation goals can be effectively applied to public health contexts with minimal modification. As public health systems worldwide respond to evolving challenges and increasing demands for data-driven decision making, structured evaluation approaches like the DHI provide an evidence-based foundation for strategic planning to advance digital transformation that can help bridge the digital divide and advance more equitable, responsive, and resilient public health services.

## Limitations

This study has several important limitations that should be considered when interpreting the findings. First, the data collection occurred in 2024, as a snapshot of the current landscape of the DHSS. At this time, the digital health capacity in Missouri’s DHSS was rapidly evolving, and organizations were varied in progress towards digital health transformation goals. For example, some organizations had already started investing in digital infrastructure which was evident by the discrepancy of analytics capacity among the DHSS teams across Missouri. The study’s cross-sectional design provides a snapshot of digital maturity at a single point in time, limiting our ability to assess how digital capabilities evolve over time, or develop in response to specific interventions. Second, there were two divisions, DA and DCR that completed only partial DHI assessments (excluding person-enabled) due to their mandate and the structure of their programs serving the public that precluded data collection on person-enabled health services. Finally, the assessment of digital maturity and capacity of State public health system is the first in the world to be achieved using the DHI tool. Thus, comparisons with other global health systems are very limited given no other country has undertaken such an assessment of their State public health system.

## Conclusion

The assessment of Missouri’s public health system reveals significant variations in digital maturity across the 59 teams that may contribute to varied approaches to delivery of public health services and population health outcomes. This first comprehensive application of the Digital Health Indicator in a state public health system demonstrates how digital capabilities can vary dramatically within the same organization. The findings highlight a pathway forward through two key strengths: exemplary programs within DHSS that have successfully achieved higher digital maturity (particularly in DCPH), and strong organizational readiness for digital advancement across the department. These strengths provide a foundation for knowledge transfer and strategic investment to build on existing strengths, and focus investments on overcoming gaps in infrastructure, workforce development, analytics capabilities, and citizen engagement tools. Beyond Missouri, this study establishes an evidence-based framework for digital health assessment in public health settings globally, offering validated metrics that enable systems to benchmark capabilities, identify opportunities to advance digital transformation, and measure progress over time. As a comprehensive assessment tool, the DHI not only evaluates current digital capabilities but also provides a strategic roadmap that helps public health systems prioritize their investments and develop targeted implementation strategies that are data driven and advance progress towards digital transformation goals. As public health systems worldwide face increasing pressure to leverage data for decision-making while ensuring equitable service delivery, structured assessment approaches like the DHI provide the evidence base needed to guide digital investments that strengthen population health outcomes and resilience of public health systems.

## Data Availability

The datasets presented in this article are not readily available because the data that support the findings of this study are owned by HIMSS and the State of Missouri. The EMRAM (Electronic Medical Record Adoption Model) data is owned by HIMSS and is not publicly available. Requests to access the datasets should be directed to Anne.Snowdon@uwindsor.ca.
